# Surgically treated rare intestinal bleeding due to submucosal hematoma in a patient on oral anticoagulant therapy: A case report

**DOI:** 10.1097/MD.0000000000013252

**Published:** 2018-11-16

**Authors:** Wei-Hua Yu, Chao Feng, Tie-Mei Han, Shun-Xian Ji, Lan Zhang, Yi-Yang Dai

**Affiliations:** a Department of Gastroenterology; b Department of Cardiology; c Department of Pathology; d Department of Radiology, The Fourth Affiliated Hospital, Zhejiang University School of Medicine, Yiwu, China.

**Keywords:** anticoagulant, colon, endoscopic ultrasound, hematochezia, intestine, submucosal hematoma

## Abstract

**Rationale:**

Bleeding in the gastrointestinal tract is a common complication of oral anticoagulant therapy (AT), and it usually appears as mucosal erosion or ulcer; however, intestinal submucosal hematoma (ISH) is an uncommon cause of hemorrhage.

**Patient concerns:**

This report presents the case of a 70-year-old woman with acute hematochezia induced by AT. She underwent computed tomography and endoscopy.

**Diagnoses:**

Colon submucosal hematoma.

**Interventions:**

Conservative treatment had no effect, and the patient underwent emergency surgery.

**Outcomes:**

Surgical resection showed hemorrhage and necrosis in the left colon, and the patient recovered 24 hours after surgery and continued AT.

**Lessons:**

The present case indicates that the ISH should be kept in mind as a complication of AT. It can be managed conservatively in some stable patients, but emergency surgery may be needed in some serious situations.

## 1 Introduction

Many clinical conditions can lead to lower alimentary tract bleeding, including tumor, ischemic bowel disease, and ulcerative colitis. Recently, with the increased use of anticoagulant therapy (AT), such as warfarin and heparin, the incidence and complications of alimentary tract bleeding have become more common.^[1]^ Generally, mucosal injury resulting from anticoagulant use is the direct cause.^[2]^ Further, submucosal hematoma is rarely reported.^[3]^ A review of related literature revealed that the most common sites are the esophagus and duodenum; moreover, small intestinal submucosal hematoma (ISH) has been reported in 1 per 2500 patients on AT each year, and colon submucosal hematomas have been described less frequently in the literature.^[3]^ The clinical symptoms of colonic submucosal hematomas typically include abdominal pain, intestinal tract obstruction, bleeding, and so on. Most ISH cases were diagnosed by endoscopy, and radiological examination, or both.^[4–7]^ Even in some cases of submucosal tumor, patients required surgical intervention for diagnosis and treatment.^[8]^ We propose that endoscopic ultrasonography (EUS) would help in the differential diagnosis of similar or atypical lesions, as, to our knowledge, this has not been reported. ISH can be treated conservatively, but surgical treatment is necessary in some patients under serious conditions or when conservative management is unsuccessful. Herein, we present a case of hematochezia arising from the colon submucosal hematoma due to AT, but EUS was not used to confirm the diagnosis because it developed rapidly and was diagnosed clearly using endoscopy and computed tomography (CT).

## 2 Case report

**Figure 1:**
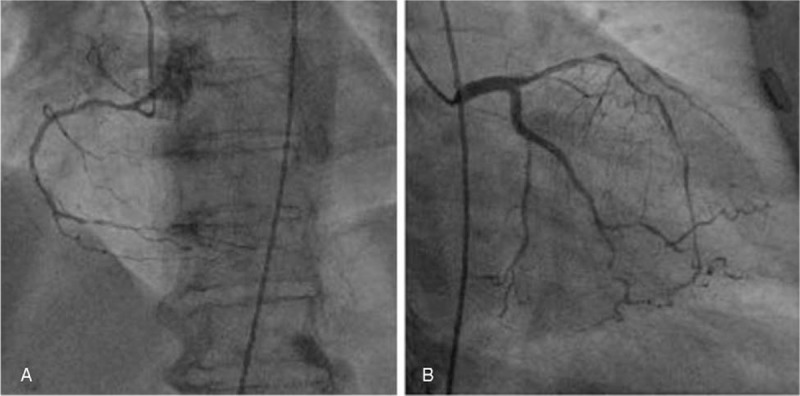
(A, B) Coronary angiography shows severe stenosis of both left and right coronary arteries.

A 70-year-old female patient was admitted to our cardiovascular department for chest distress for 11 hours. The patient presented with shortness of breath at rest and difficulty in laying in the recumbent position. She had been diagnosed with hypertension and diabetes mellitus several years prior and was on oral medication for their treatment. After clinical examination, she was diagnosed with coronary atherosclerotic heart disease and subsequently underwent coronary angiography and stent implantation (Fig. 1A and B), with heparin (total 5500 units, including arterial intrathecal injection of 2000 units and intravenous injection of 3500 units) administration during the operation. After coronary angiography, she was prescribed aspirin (100 mg per day), TiGraylo (90 mg once every 12 h), and enoxaparin sodium (0.4 mL once every 12 h subcutaneously) for 3 days, and her chest distress and shortness of breath had relieved. On the third day after the operation, she was diagnosed with hematochezia without abdominal pain.

**Figure 2:**
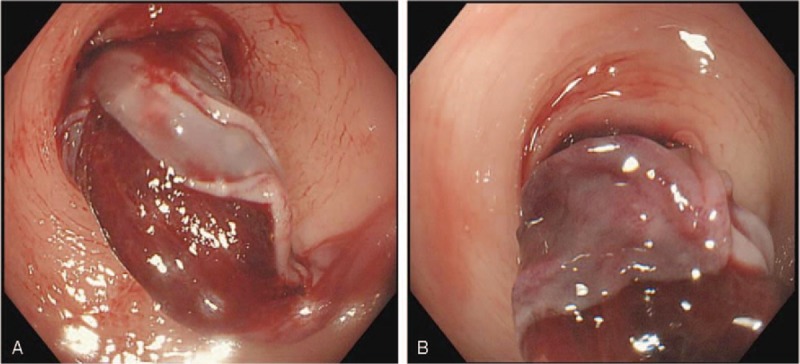
(A, B) Submucosal hematoma ruptured into the intestinal cavity, and the intestinal cavity was stenosed.

**Figure 3:**
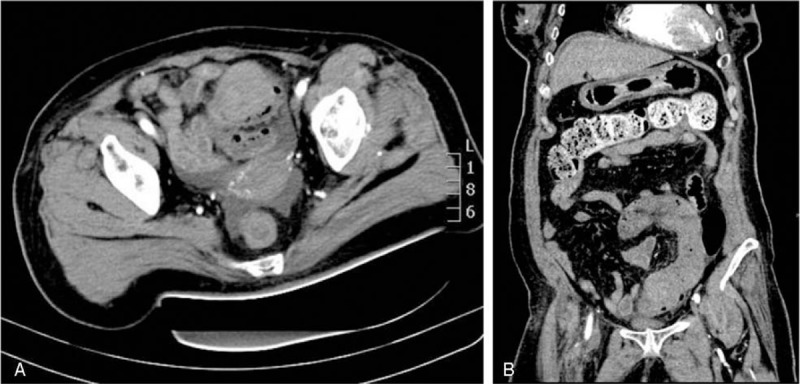
(A, B) Plain computed tomography showed hyperdense lesion in the intramural aspect of the sigmoid colon.

**Figure 4:**
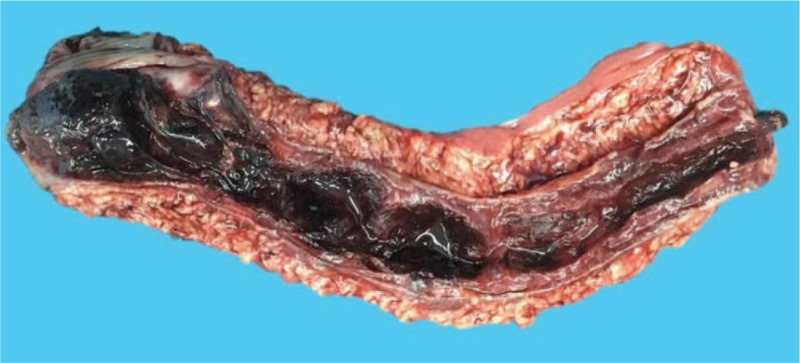
Macroscopically, the size of the lesion was 60 mm × 38 mm × 20 mm and the cut surface appeared solid and yellow with focally reddish patches.

**Figure 5:**
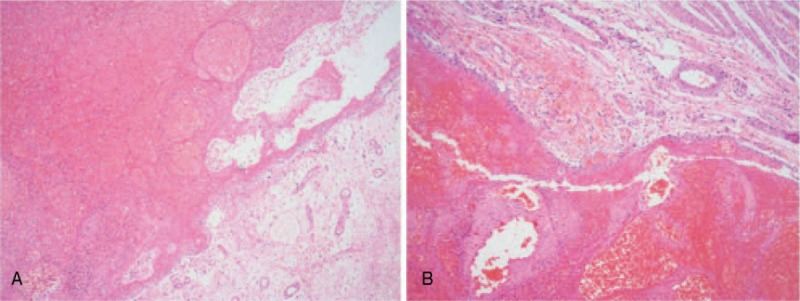
(A, B) The mucosal membrane was replaced by numerous hemorrhagic and necrotic tissues, the submucosa was edematous, and the muscle layer was intact, showing bleeding in some muscles (hematoxylin and eosin staining, × 40).

Physical examination revealed mild abdominal tenderness, especially on the left lower quadrant, with signs indicative of peritoneal irritation. A neoplasm-like mucous clot connected to the bowel was found in the anus. Laboratory tests showed declined levels of hemoglobin (102 g/L), prolonged prothrombin time (13.4 s; normal range 9.8–12.3), and an international normalized ratio of 1.2 (normal range 0.9–1.1). Colonoscopy revealed a submucosal mass, covered with a partially ruptured mucous membrane and with some clots in the cavity, which caused obstruction in the sigmoid colon, hindering further examination (Fig. 2A and B). Abdominal CT showed submucosal hematoma in the sigmoid colon and the density of the mass did not significantly change on enhanced CT scan. The left flexure of the colon was thick, indicating that it could also be a hematoma (Fig. 3A and B). Clinical symptoms did not resolve with conservative treatment, which included anticoagulant cessation, total parenteral nutrition, and blood transfusions. Two days later, the patient complained of fever, body temperature of 38.8°C, with aggravated lower abdominal pain, and abdominal distention, and physical examination revealed peritoneal irritation. The patient underwent emergency exploratory surgery, which revealed a huge submucosal hematoma in the sigmoid colon and a necrosed bowel, 40 cm in length, with a clear boundary. Sigmoidectomy and end colostomy were performed. Pathological examination showed left-half colonic hemorrhage with necrosis (Figs. 4 and 5A, B). Considering that the patient had a high risk for developing an embolism 24 hours after the surgical intervention, she was prescribed enoxaparin sodium (0.4 mL once daily, subcutaneous injection). Six and 9 days later, clopidogrel (75 mg per day) and aspirin (0.1 g per day) were added, respectively, and enoxaparin sodium administration was stopped. The patient was administered the same 2 antiplatelets until May 2018, after which she was prescribed oral clopidogrel bisulfate (50 mg per day) and aspirin (0.1 g per day). She did not have intestinal bleeding and occult blood test was negative on the latest follow-up.

Patient consent was obtained, and approval for the study was granted by the ethics committee of the Fourth Affiliated Hospital, Zhejiang University School of Medicine.

## 3 Discussion

As life expectancies increase, cardiovascular and cerebrovascular diseases are becoming more common, and the incidence of anticoagulant administration and the relative complications of anticoagulants are increasing. The most serious complication of anticoagulant use is bleeding,^[1]^ and the gastrointestinal tract is the most common site of anticoagulant drug-related injury, where mucosal erosion or ulcers are generally observed as complications.^[2]^ After the first report on ISH by McLauchlan in 1838,^[9]^ it was reported upon very rarely.

ISH mostly occurs in the small intestine and rarely in the colon.^[3]^ Except for AT, the pathologic process can be induced by abdominal trauma,^[10]^ blood dyscrasias^[11]^ iatrogenic cause,^[4]^ and so on. The present case showed acute hematochezia due to ISH. After careful medical history taking, examinations, and without special endoscopic operation before colon bleeding, the patient was administered anticoagulant treatment; thus, we speculated that the medication was the most likely cause of ISH. According to the literature,^[6,7,12–15]^ antiplatelet and anticoagulant drugs cause bleeding; however, the latter drug is more likely to cause bleeding. With the medications taken before the intestinal bleeding and after surgery, we considered that the culprit medicine was heparin. The duration of use of antiplatelet or anticoagulant drugs before onset was 1 day to 5 years. The drug used for the shortest time was heparin,^[6,7,12–15]^ which is injected during and after surgery, similar to that in the present case. However, the exact pathogenesis remains unclear. The proposed mechanism for ISH is related to the broken continuity of terminal arteries at points of penetration of the lamina propria of the alimentary tract.^[16]^ Increased bleeding tendency because of antiplatelet or AT is considered to be involved in the pathogenesis of submucosal hematoma.^[17]^ The hematoma forms within the muscular layers and expands until its growth is limited by adjacent structures, which seldom causes hemorrhage into the mediastinum or peritoneal cavity.^[18]^ In this case, the patient received antiplatelet and AT, which could have aggravated the risk of ISH.

In most cases of ISH, the clinical symptoms are abdominal pain, abdominal distention, vomiting, and hematochezia, and physical examination revealed abdominal tenderness, either localized or diffused, abdominal guarding in varying degrees, peritoneal irritation, or bowel obstruction. In this case, the patient had hematochezia, abdominal pain, and abdominal distention, and on physical examination, as disease progressed, the abdominal tenderness developed into peritoneal stimulation.

The prothrombin time and international normalized ratio are usually abnormally prolonged,^[3]^ but in some cases, they are normal or within the therapeutic range.^[6,19]^ Although a wide range of diagnostic imaging techniques is recommended, including barium enemas, ultrasonography, and CT, they were all categorized as nonspecific tests. Barium enemas usually show characteristics of “picket fence” and “coiled-spring” signs. On ultrasonography, masses in the intestinal wall appear as round or non-peristaltic tubular masses with a central echogenic core of compressed mucosa surrounded by an anechoic halo that corresponds to the bowel wall thickened by infiltration of hemorrhage.^[18]^ Ultrasonography showed 71.4% sensitivity for detecting such findings. CT, usually with 80% to 100% sensitivity rate, showed characteristic features,^[6]^ such as circumferential bowel wall thickening, intramural hyperdensity with Hounsfield units characteristic for blood (30–80 H), luminal narrowing, and intestinal obstruction.^[20]^ Moreover, 2 additional signs were commonly seen in intramural hematoma: “coiled spring” and “pseudokidney” signs.^[21]^ Endoscopically, it presents as blue submucosal mass with or without a visible tear, and when the mucous membranes covering the hematoma were peeled off, a shallow ulcer formed over a wide area with scars appearing after about 1 month.^[22]^ If the patient is stable, but with an unclear diagnosis, a pathological finding may help differentiate between a hematoma and a neoplasm, but a colonoscopic biopsy may result in bleeding. A previous study reported that this disease was misdiagnosed as neoplasm or false aneurysmal tumor, and a final diagnosis was obtained by laparotomy.^[8]^ Recently, EUS has emerged as a useful diagnostic tool that facilitates determination of the thickness of the wall from which the lesion has originated. In addition, the technique facilitates the assessment of echostructures of the lesion and the thickness of the infiltration, and it enables the physician to perform thin-needle or core-needle biopsy to collect materials for cytological and histopathological analyses.^[23]^ In the present case, abdominal CT revealed bowel wall thickening and hyperdensity in the luminal area, whereas endoscopy showed that the hematoma ruptured into the bowel cavity with stenosis. Moreover, the pathological examination excluded any possibility of malignancy, so we diagnosed the case as ISH.

Normally, conservative management is considered the first-line therapy, which constitutes immediate cessation of any anticoagulant, that is, correcting coagulation parameters by infusing fresh-frozen plasma and vitamin K, followed by bowel rest and total parenteral nutrition. However, the timing of starting this treatment (between 48 h and 2 months) was not described clearly.^[6,7,12–15]^ Surgical intervention should be reserved for those who exhibit either a deteriorating condition or an unrelenting intestinal obstruction or for those with signs of bowel necrosis or peritonitis. Recently, several new therapeutic strategies, as alternatives to surgical treatment, have been reported, such as percutaneous ultrasonically guided drainage and balloon dilatation using endoscopic incision and drainage.^[24]^ However, they pose a risk for intestinal perforation. Ulla-Rocha et al^[25]^ reported 2 cases of perigastric hematoma and perirectal hematoma, which were treated successfully with endoscopic ultrasound-guided fine needle aspiration (EUS-FNA)-guided drainage and stent placement, and they recommended it as a safe method for chronic hematoma.^[26]^ To our knowledge, ISH treated with EUS-FNA had not been described previously. As “tamponade effect” could lead to further bleeding,^[10]^ the exact duration and time of drainage should be further evaluated. In this case, self-drainage was performed after the mucous membrane ruptured into the intestinal cavity, but as conservative treatment was not successful and there were signs of bowel necrosis, emergency surgery was required. We presumed that this condition emerged because of the delayed correction of coagulation by transfusing fresh-frozen plasma and vitamin K.

In conclusion, AT can lead to ISH, and the associated clinical symptoms can be diagnosed with barium enema, ultrasonography, abdominal CT, or endoscopy. Further, EUS or EUS-FNA can be performed to differentiate diagnosis of some similar or atypical lesions. Conservative management and surgical intervention are traditional treatment strategies. To avoid unnecessary surgery, EUS and EUS-FNA procedures should be recommended for unresolved hematoma or untreated intestinal obstruction in patients with stable vital signs.

## Author contributions

**Conceptualization:** Wei-Hua Yu.

**Data curation:** Wei-Hua Yu, Chao Feng, Tie-Mei Han, Shun-Xian Ji, Lan Zhang.

**Formal analysis:** Yi-Yang Dai.

**Investigation:** Wei-Hua Yu.

**Methodology:** Wei-Hua Yu.

**Supervision:** Wei-Hua Yu, Yi-Yang Dai.

**Validation:** Wei-Hua Yu.

**Writing – original draft:** Wei-Hua Yu.

**Writing – review & editing:** Wei-Hua Yu.
